# HIV/AIDS stigma-associated attitudes in a rural Ethiopian community: characteristics, correlation with HIV knowledge and other factors, and implications for community intervention

**DOI:** 10.1186/1472-698X-12-6

**Published:** 2012-05-03

**Authors:** Alan R Lifson, Workneh Demissie, Alemayehu Tadesse, Kassu Ketema, Randy May, Bereket Yakob, Meka Metekia, Lucy Slater, Tibebe Shenie

**Affiliations:** 1 Division of Epidemiology and Community Health, University of Minnesota, 1300 S. Second Street, Suite 300, Minneapolis, MN 55454-1015, USA; 2 Ethiopian Office, National Alliance of State and Territorial AIDS Directors, Addis Ababa, Ethiopia; 3 World Health Organization Country Office for Ethiopia, Addis Ababa, Ethiopia; 4 Regional Health Bureau, Southern Nations, Nationalities and People’s Region, Hawassa, Ethiopia; 5 Global Program, National Alliance of State and Territorial AIDS Directors, Washington, DC, USA

## Abstract

**Background:**

Whether scale-up of HIV prevention and care will reduce negative attitudes and discriminatory practices towards persons living with HIV/AIDS (PLWH) is uncertain. An HIV knowledge and attitude survey was conducted in a rural Ethiopian community where HIV prevention and treatment was being rapidly scaled up. Data were analyzed to identify prevalence of and factors associated with stigma-associated attitudes towards PLWH.

**Methods:**

We surveyed 561 adults from 250 randomly selected households in the rural town of Arba Minch and surrounding villages about positive or negative attitudes towards PLWH, as well as demographic characteristics, and knowledge about HIV transmission and treatment.

**Results:**

Eighty percent of respondents agreed with ≥ 1 negative statements indicating blame or shame towards PLWH and 41% agreed with ≥ 1 negative statements associated with distancing themselves from PLWH. However, only 14% expressed negative responses about whether PLWH should receive support from their communities. In multivariate analysis, a greater number of negative attitudes towards PLWH was significantly (p < 0.05) associated with: female gender (Odds Ratio [OR] = 1.51), living in a rural village (vs. town neighborhood) (OR = 3.44), not knowing PLWH can appear healthy (OR = 1.78), lack of knowledge about perinatal transmission (OR = 1.49), lack of knowledge about how HIV is not transmitted (e.g. casual contact) (OR = 2.05), lack of knowledge about HIV treatment (OR = 1.80), and not personally knowing a PLWH (OR = 1.41).

**Conclusions:**

In a rural Ethiopian setting in which rapid scale-up of HIV treatment occurred, many respondents still characterized HIV as associated with shame or blame, or indicated PLWH would be isolated or discriminated against. HIV stigma can hamper both prevention and treatment programs. We identified multiple issues which, if addressed, can help promote a more positive cycle in which PLWH are appreciated as members of one’s own community who are affirmatively interacted with and supported. Stigma reduction programs should address knowledge gaps such as fears of casual contact contagion, and lack of awareness of medical interventions to help prevent HIV disease, as well as building upon community-based attitudes of the importance of supporting and showing compassion for PLWH.

## Background

Despite international progress in scaling up HIV prevention and treatment efforts, negative attitudes and discriminatory practices towards persons living with HIV/AIDS (PLWH) represent a major and persistent barrier [1-17]. Stigma can occur at the community level, in institutions (e.g., healthcare facilities), and among PLWH themselves (internalized stigma). At the community level, stigma may include negative judgments about PLWH (e.g., blame or shame) and distancing (enacted stigma) practices, such as exclusion from social events or abandonment. For PLWH, fear of stigma and discrimination may be major barriers towards: seeking HIV testing to learn one’s status, participation in prevention of mother to child transmission (MTCT), programs, disclosure of HIV status to spouses or others, linkage to and retention in HIV treatment, and adherence to antiretroviral therapy (ART) [1-15].

HIV stigma has been reported from many countries of sub-Saharan Africa (SSA) [1,3,6,8-12,14-18], where there are over 22 million PLWH, and where in 2009 alone there were 1.8 million new HIV infections and 1.3 million HIV deaths [19]. It has been suggested that in an era in which ART and other HIV care is becoming increasingly accessible, and HIV becomes viewed as a manageable disease, stigma and discrimination may reduce [5,6,8,16,20]. However, studies evaluating countries in SSA where ART has been scaled up have reached varying conclusions about the impact on HIV stigma [6,16,18].

Ethiopia, with an estimated 1.1 million PLWH [21] reflects these challenges for SSA. A survey in Ethiopia and other East African countries during 2001–2003 (before ART became available) reported significant stigma at the community level, with contributing factors including an incomplete knowledge about HIV transmission (especially fear of casual contact), shame and blame associated with HIV as sexually transmitted, and fear of death [15]. In 2005, Ethiopia launched a program to provide free ART, with decentralization and rapid scale-up of HIV care; in 2009, ART was provided to an estimated 53% of those with advanced HIV infection [21]. Education efforts include improving the percentage of the population who correctly understand ways of preventing HIV and reject major misconceptions about HIV transmission. The government has also been working closely with civil society to monitor stigma and discrimination towards PLWH, and to design programs to raise awareness and understanding.

Approximately 82% of Ethiopians live in rural areas [22], and available data suggested that HIV prevalence may be increasing in small market towns compared to large cities, with these smaller towns serving as bridging sites for urban to rural spread of HIV [21]. Other countries in SSA also face the challenge of rural HIV; more than two-thirds of the population in the 25 African countries most-affected by AIDS lives in rural areas [23]. Compared to those in urban areas, rural populations may have lower HIV knowledge and awareness levels, more difficulty accessing HIV testing and care, and greater challenges adhering to HIV treatment [23-27].

In preparation for a community support program for PLWH in rural southern Ethiopia, we conducted in 2010 a general population survey of HIV knowledge and attitudes towards PLWH; the study was conducted in an area in which HIV care and treatment activities were being rapidly scaled up. We determined the prevalence of negative attitudes towards PLWH, the level of knowledge about HIV transmission and treatment, and the correlation of negative attitudes with demographic characteristics and specific knowledge areas.

## Methods

### Study setting

Arba Minch is a rural town located in the foothills of the Rift Valley wall in southern Ethiopia. In addition to the town, many live in the surrounding villages and support themselves through farming. As of February 2010, 2,886 patients were enrolled in HIV care at Arba Minch Hospital, the main zonal referral hospital, and 1,849 had been started on ART, more than double the numbers from 2007; during the same three years, the number of HIV patients enrolled in care at the Arba Minch Health Clinic (a local clinic serving primarily the town) increased over eight-fold [28,29].

The smallest and one of the most important units of administration in Ethiopia is the kebele, based in a village or neighborhood; kebeles are responsible for many basic municipal services. Every resident of Ethiopia belongs to a kebele, and it is a strong focus of identification. Ten kebeles were selected from Arba Minch town and the surrounding villages for this survey: four kebeles were neighborhoods within Arba Minch town and six kebeles were in outlying villages. The main criterion for selection was prior knowledge that there were a large number of PLWH in that kebele; this was based on summary data on residence for patients enrolled in Arba Minch Hospital’s HIV Clinic, plus information from local PLWH associations.

### Identification of sample households and participant recruitment

Kebele administrations maintain a listing of all households, identified by family name; the mean number of households per kebele was 532 in the four town kebeles, and 704 in village kebeles. Using this list, for each kebele, 25 households were selected using a random number generator.

Interviewers approached each selected household and explained the study, indicating they would like to privately interview all adults living in that household. Inclusion criteria included age ≥18 years and being a permanent resident of that kebele. The decision to interview all household adults was made so that in addition to the “head of the household”, we would also get adequate representation of women and younger adults. To reach all adults, interviewers sometimes had to visit households on several occasions, including evenings or early mornings. If all adults in a household refused to be interviewed, or if the household could not be located, another household from the same kebele was randomly selected.

Of 250 households selected in the first randomization, in 34 (14%) the family moved or otherwise could not be located (21% in town neighborhoods and 9% in villages); 34 additional households were then chosen. Of 638 adult residents of these households who met inclusion criteria, 47 (7%) had temporarily moved away (such as for school or work) or were otherwise unavailable; of the remaining 591, 95% agreed to be interviewed. In only four households did all adults refuse participation.

Data collection occurred in 2010 by 24 graduate students or graduates of Arba Minch University, after a two-day pre-survey training. Surveys were conducted privately and administered verbally in Amharic. Survey items included: (a) demographic characteristics (e.g., age, gender, education, marital status); (b) positive and negative attitudes towards PLWH; and (c) knowledge about HIV transmission and HIV treatment.

Attitude and knowledge items were chosen after reviewing a number of published reports and validated questionnaires, and selected to reflect certain parameters of particular interest. Questions about attitudes towards PLWH were selected from three categories: (1) negative attitudes of blame or shame towards PLWH; (2) negative discrimination or exclusion practices towards PLWH; or (3) positive feelings of compassion towards PLWH and responsibility to support them. Questions on HIV knowledge were from five categories: (1) whether one could have HIV and appear healthy; (2) sexual HIV transmission and its prevention; (3) MTCT and its prevention; (4) how HIV is not transmitted (especially through casual contact); and (5) knowledge and benefits of HIV treatment, especially ART. Within each knowledge category, we evaluated the number of correct responses.

### Analysis

We created an overall stigma score, assigning one point for each agreement with a negative attitude statement, and one point for each disagreement with a positive attitude statement. Respondents were dichotomized based upon the median stigma score. Univariate analyses with chi-square for categorical variables and Student’s *t*-test for continuous variables were conducted to identify factors associated with an increased number of negative attitudes towards PLWH. Multivariate analysis was conducted using a generalized linear model with the GENMOD procedure in SAS [30], controlling for kebele of residence (since respondents within a kebele may be more similar than between kebeles).

### Ethics

This study was reviewed and approved by the Institutional Review Boards of the University of Hawassa and the University of Minnesota. All study subjects provided informed consent, and data collectors were trained in informed consent procedures; household members who could not provide consent because of physical or mental illness were excluded. The project was also presented to kebele leaders, and permission was sought to work in the kebele.

## Results

### Study population

Of 561 participants, 65% were from rural villages and 35% from town neighborhoods. Fifty-three percent were female; the median age was 30 years (interquartile range [IQR] = 22, 44 years). Sixty-two percent were married and 32% single; the remainder were widowed or separated. The median number of adults and children living in a household was 5 (IQR = 4,8). In terms of highest level of education, 49% either never attended school or attended some primary school but had not completed it.

### Negative attitudes towards PLWH

Responses to individual questions about attitudes towards PLWH are summarized in Table 1. Eighty percent responded with one or more negative responses associated with blame or shame towards PLWH, the most common (74%) being that HIV was God’s punishment for bad behavior. Forty-one percent responded with one or more negative responses associated with distancing themselves from PLWH, the most common being that they would not buy vegetables in the market from a person with AIDS (27%). On the other hand, only 14% expressed one or more negative responses about whether PLWH should receive support from their communities; 92% said the community should help socially support them. Finally, 60% said they personally knew someone with HIV/AIDS.

**Table 1:** Attitudes about people living with HIV/AIDS, community residents of kebeles, Arba Minch, Ethiopia

Blame or Shame:	**Yes**	**No**	**DK**
HIV is a punishment from God for bad behavior	74%	22%	4%
People with HIV should be ashamed of themselves	36%	62%	2%
I would be ashamed to be seen in public with friend known to have AIDS	29%	71%	1%
Distancing:			
I would not buy vegetables in the market from someone who had AIDS	27%	71%	2%
PLWH in our kebele would not be welcome in weddings or parties	15%	85%	< 1%
PLWH in our kebele would probably be abandoned or sent away by their spouse or family	18%	81%	1%
Support for PLWH:			
If PLWH in our kebele, community should help socially support them	92%	6%	1%
If PLWH in our kebele, community should share responsibility for their care and treatment	87%	10%	3%
PLWH deserve our compassion and support	94%	4%	3%

Abbreviations: PLWH: Person living with HIV/AIDS; DK = Don’t know.

### Knowledge about HIV

Responses to individual knowledge questions about HIV are summarized in Table 2. The percent correctly answering all questions in each category were as follows: 70% for questions about whether one could have HIV and appear healthy; 68% for questions about sexual transmission, 40% for questions about MTCT, 54% for questions on how HIV is not transmitted, and 38% for questions about ART/HIV treatment.

**Table 2:** Knowledge about HIV transmission and treatment, community residents of kebeles, Arba Minch, Ethiopia

HIV Infection vs. Disease:	**Yes**	**No**	**DK**
A healthy looking person can have HIV	80%	14%	6%
Only people who look sick can spread HIV	15%	82%	3%
Sexual Transmission:			
Can get HIV by having sex with an infected person	95%	3%	2%
Can protect against HIV by having one uninfected faithful sex partner	91%	8%	1%
Can protect against HIV by using a condom correctly and consistently	77%	11%	12%
Mother-to Child Transmission:			
A woman with HIV can give HIV to baby during pregnancy or delivery	70%	20%	10%
A woman with HIV can give HIV to her baby by breast feeding	84%	9%	7%
There are ways a pregnant woman with HIV can reduce the chance of giving HIV to her unborn child	55%	20%	25%
Ways HIV is Not Spread:			
Can get HIV from mosquito bites	26%	66%	8%
Can get HIV by sharing a meal with PLWH	11%	88%	2%
Can get HIV by touching PLWH	7%	91%	2%
Can get HIV by sleeping in same room as PLWH	10%	87%	3%
My child could get HIV by playing with another child who had AIDS	12%	86%	1%
HIV Treatment:			
If you get AIDS, there is nothing you can do to help keep you alive	26%	68%	6%
There are treatments that prolong the life of PLWH	86%	7%	7%
There is a cure for AIDS	23%	72%	5%
If PLWH gets started on ART, OK to stop once you feel better	12%	80%	8%
If PLWH gets started on ART, it is important to take every day to prevent them from becoming sick	91%	3%	6%

Abbreviations: PLWH: Person living with HIV/AIDS; ART = antiretroviral therapy; DK = Don’t know.

Of note, although 91% believed having one faithful uninfected sex partner was protective, 23% either did not know or did not believe that condoms correctly and consistently used could provide protection. Thirty percent did not know that HIV could be transmitted during pregnancy or delivery, and 45% were unaware there are ways to prevent perinatal transmission; among women, 33% and 51% did not respond correctly to these questions. Reflecting some uncertainty about treatment for HIV, although 86% of respondents agreed there are treatments that could prolong the life of PLWH, 26% believed that there was nothing someone with AIDS could do to help keep them alive and 23% believed there was a “cure” for AIDS; in response to an open-ended follow-up question, some of the most common “cures” included “prayer”, “faith” and “holy water”. When asked if they felt they had enough information about HIV/AIDS, 54% responded “no”.

### Determinants of negative attitudes towards PLWH

On the nine attitude questions, 80 (14%) expressed no negative responses, 278 (50%) 1–2 negatives, 133 (24%) 3–4 negatives, and 70 (12%) ≥5 negatives (median =2). When evaluated as continuous variables, there was a high negative correlation between the number of negative attitudes expressed and the number of correct responses on the 18 knowledge questions (r = −0.465, p < 0.001).

Variables tested for their association with a higher stigma score (defined as ≥3 negative responses) are summarized in Table 3. In univariate analysis, a higher stigma score was significantly (p < 0.05) associated with the following characteristics: female gender, lower education level, and living in a village (vs. town) kebele. Age group was not associated with stigma; when evaluated as a continuous variable, the mean ages were 35.5 years for high stigma and 34.1 for lower stigma (p > 0.10). Higher stigma was significantly associated with giving one or more incorrect responses on the following knowledge areas: HIV infection vs. disease, sexual transmission, MTCT, ways HIV is not spread, and HIV treatment. Those who personally knew a PLWH were significantly less likely to have a high stigma score.

**Table 3:** Multivariate analysis for demographic and knowledge factors associated with a high stigma score, community residents of kebeles, Arba Minch, Ethiopia

**Characteristic**	**Number (%) with**	**Multivariate analysis**	
	High stigma score*	**p-value**	**Odds ratio**
Gender		0.041	
Male	72 (28%)		1.00 (Referent)
Female	130 (44%)		1.51 (1.02, 2.23)
Age		0.081	
≤ 21 years	46 (38%)		0.57 (0.32, 1.01)
22–44 years	97 (33%)		0.75. (0.55. 1.02)
≥ 45 years	58 (42%)		1.00 (Referent)
Education		0.051	
Completed primary school	58 (20%)		1.00 (Referent)
Didn’t complete primary school	145 (53%)		2.15 (0.998, 4.62)
Residence		< 0.001	
Town neighborhood kebele	36 (18%)		1.00 (Referent)
Rural village kebele	167 (46%)		3.44 (2.16, 5.50)
Knowledge about infection vs. disease		0.042	
Correctly answers all items	110 (28%)		1.00 (Referent)
Incorrect, one or more items	93 (56%)		1.78 (1.02, 3.08)
Knowledge about sexual transmission		> 0.10	
Correctly answers all items	111 (29%)		1.00 (Referent)
Incorrect, one or more items	92 (51%)		1.30 (0.88, 1.93)
Knowledge about MTCT		0.014	
Correctly answers all items	55 (25%)		1.00 (Referent)
Incorrect, one or more items	148 (44%)		1.49 (1.09, 2.06)
Knowledge about ways HIV not spread		< 0.001.	
Correctly answers all items	65 (22%)		1.00 (Referent)
Incorrect, one or more items	138 (53%)		2.05 (1.40, 3.01)
Knowledge about HIV treatment		0.046	
Correctly answers all items	49 (23%)		1.00 (Referent)
Incorrect, one or more items	154 (45%)		1.80 (1.01, 3.21)
Personally knows PLWH		0.020	
Yes	88 (27%)		1.00 (Referent)
No	108 (50%)		1.41 (1.06, 1.89)

* defined as ≥3 negative responses.

Abbreviations: PLWH: Person living with HIV/AIDS; ART = antiretroviral therapy.

In a multivariate model which included all factors, seven were significantly (p < 0.05) associated with a high stigma level: female gender, living in a rural village (vs. town neighborhood), not knowing PLWH can appear healthy, lack of knowledge about MTCT, belief in or uncertainty about ways HIV is not transmitted, lack of knowledge about HIV treatment, and not personally knowing a PLWH (Table 3); education level (whether or not at least completed primary school) and age group were of borderline significance.

## Discussion

In a rural town and surrounding villages where HIV care and treatment is being rapidly scaled up, we found that components of HIV stigma are still common. Those who had more negative attitudes about PLWH were more likely to believe that HIV can be spread through casual contact or mosquitoes, and to lack knowledge about the natural history of HIV, as well as ART and its benefits. Personally knowing a PLWH was associated with fewer negative attitudes.

Health related stigma has been characterized as a process of social disqualification of individuals and populations identified with particular health problems, associated with exclusion, rejection, blame or devaluation [31]. Stigma can reflect and perpetuate existing inequalities and marginalization of socially disenfranchised groups [32].

Stigma was recognized early in the AIDS epidemic as a barrier to HIV prevention, treatment and support. Manifestations of HIV stigma include: negative judgments about PLWH (such as shame or blame-associated judgments); avoidance or discriminatory behaviors (enacted stigma) in family, community or institutional settings; discriminatory laws or policies; and self (internalized) stigma.

The focus of this analysis was HIV stigma in the community setting. Ethiopia, like many countries in SSA, is strongly grounded in the belief, especially in rural settings, that what happens to one person concerns the whole community; therefore, negative judgments and exclusion by the community can have devastating effects [1,33].

In terms of attitudes towards PLWH, we found both positive and negative beliefs. On the one hand, 80% agreed with one or more negative judgments about PLWH that reflected shame or blame, and 41% agreed with actions reflecting distancing from PLWH. On the other hand, over 85% felt that PLWH deserved compassion and support from their communities. This seeming contradiction has been previously noted in Ethiopia and other African countries [15]. Even when PLWH are believed to have morally transgressed, a strong sense of community responsibility can lead people to believe that PLWH should still be cared for and treated with compassion [15,17].

Our finding of a significant correlation between HIV knowledge and negative attitudes towards PLWH is consistent with other studies linking poor knowledge about HIV/AIDS (including HIV transmission) with increased HIV stigma [2,3,34,35]. Knowledge gaps associated with stigma in our analysis included an incomplete understanding about how HIV is not transmitted (especially through casual contact), the natural history of HIV infection (which can initially be asymptomatic), ART (including its potential benefits), and MTCT (including prevention of perinatal transmission). It is concerning that 33% of women did not know that HIV could be transmitted perinatally, and that 51% were unaware that there are ways to reduce MTCT. In 2009, only 35% of all pregnant African women received an HIV test, and only 54% of HIV-positive women received ART to prevent MTCT [36].

Although knowledge about sexual transmission was not statistically associated with stigma, it is concerning that although over 90% knew that HIV could be transmitted sexually and that having one uninfected faithful partner was protective, 23% were uncertain or did not believe that people could protect themselves from HIV by consistent and correct condom use. Lack of awareness or belief in the benefits of condom use jeopardizes one important strategy in a comprehensive HIV prevention program [37,38].

In our analysis, personally knowing a PLWH was a significant predictor of lower stigma. Other studies have also found that personal acquaintance with a PLWH is associated with less expressed stigma [6,35]. This supports the involvement of PLWH in both developing and implementing community-based stigma and discrimination-reduction efforts [2,5,6], and the importance of PLWH being willing to disclose their status to others.

Our findings of greater negative attitudes among those with lower education are consistent with other studies [35]. Our findings are also consistent with other studies in suggesting that stigma may manifest differently in men and women [1,17,33]. Finally, the presence of greater stigma among those living in rural villages is of particular note. Although rural HIV seroprevalence in Ethiopia is lower than in urban settings, over 80% of the Ethiopian population is rural; in 2007, it was estimated that 38% of all PLWH were rural [39].

Although the focus of this analysis was on stigma in the community setting, stigma may occur in other settings such as health care facilities, may be reflected in laws and policies of governments and other institutions, may be internalized as well as external, and may overlap with additional stigma against marginalized groups such as sex workers or men who have sex with men. This supports the need to evaluate in each local setting how HIV stigma and discrimination are manifested, so that programs to combat it can be most effectively targeted.

There are several cautions in interpreting our results. Although interviewers were trained to ask questions in a nonjudgmental fashion, some participants may have given responses they considered socially desirable, rather than reflecting how they actually felt; if so, the degree of stigma may have been greater than we identified. Second, by design, this survey was conducted in kebeles with larger numbers of PLWH; communities with few or no HIV-infected residents may have had different responses. Third, survey responses will likely differ in other counties, as well as Ethiopian regions with a different cultural, religious or social make-up; results therefore cannot be directly generalized to all other sub-Saharan African rural communities. However, as described above, our finding concerning both the nature and determinants of stigma are consistent with studies from many other countries in SSA.

## Conclusions

Knowing the prevalence and correlates of HIV-related stigma and discrimination represents an important and evidence-based component of “knowing your epidemic” to help guide HIV program efforts [2]. In a rural Ethiopian setting in which rapid scale-up of HIV treatment occurred, many respondents still characterized HIV as associated with shame or blame for bad behavior, or indicated that PLWH would be isolated or discriminated against.

Our results suggest that the best strategies to reduce such stigmatizing attitudes and practices at the community level towards PLWH will be multifaceted. Education programs need to address knowledge gaps such as fears of casual contact contagion, and lack of awareness of medical interventions to help prevent HIV disease. A variety of training techniques may be utilized, each being clear and appropriate for the target populations (including educational level, gender and cultural background), with opportunities to ask questions and engage in interactive discussion; the Ethiopian community conversations program is built on such a model [21].

Although necessary, providing such information about HIV may not be sufficient. Stigma reduction measures need to build upon the community-based attitudes identified in our survey of the importance of supporting and showing compassion for PLWH. This includes helping persons better understand stigmatizing attitudes and practices in their own community, and its harmful effects on PLWH. Religious and other opinion leaders, as well as PLWH, can play key role in such efforts.

The negative cycle of stigma prevents PLWH from learning and disclosing their status to their friends, family and community; our results indicate that lack of interaction with identified PLWH only furthers such stigma. We identified a number of issues which, if addressed, offer a changed path towards a positive cycle in which PLWH are recognized as members of one’s own community who are affirmatively interacted with and supported.

## Competing interests

There are no competing interests.

## Authors’ contributions

AL was lead on planning and coordinating the data collection, conducting analysis, and drafting the manuscript. All co-authors were involved in developing various aspects of the study protocol, including procedures for participant recruitment, as well as development of the questionnaire. WD, AT, RM, BY, MM and TT participated in implementation of this study, including training and supervision of data collectors, and communication with the kebeles in which this study took place. All coauthors provided feedback and recommendation on the interpretation of data collected in this study, Coauthors also offered valuable input and suggestions on draft versions of this manuscript, and all authors approve the final manuscript.

## Pre-publication history

The pre-publication history for this paper can be accessed here:

http://www.biomedcentral.com/1472-698X/12/6/prepub
